# Motivating non-physician health workers to reduce the behavioral risk factors of non-communicable diseases in the community: a field trial study

**DOI:** 10.1186/s13690-023-01047-w

**Published:** 2023-03-10

**Authors:** Mehran Asadi-Aliabadi, Seyed M. Karimi, Fariba Mirbaha-Hashemi, Arash Tehrani-Banihashemi, Leila Janani, Ebrahim Babaee, Marzieh Nojomi, Maziar Moradi-Lakeh

**Affiliations:** 1 Preventive Medicine and Public Health Research Center, Psychosocial Health Research Institute, Iran University of Medical Sciences, Tehran, Iran; 2 Health Sciences Research Center, Mazandaran University of Medical Sciences, Sari, Iran; 3 Department of Health Management & System Sciences, School of Public Health & Information Sciences, University of Louisville, Louisville, KY USA; 4 Department of Community and Family Medicine, School of Medicine, Iran University of Medical Sciences, Tehran, Iran; 5 Department of Biostatistics, School of Public Health, Iran University of Medical Sciences, Tehran, Iran; 6 Clinical Trial Center, Tehran University of Medical Sciences, Tehran, Iran

**Keywords:** NCDs risk factors, Randomized field trial, Non-physician health worker, Evidence-based training, Performance-based financing

## Abstract

**Background:**

Non-communicable diseases behavioral risk factors can be improved if effective interventions are designed considering the health system’s capabilities and local resources. This study evaluated the effectiveness of interventions that aimed at increasing non-physician community health workers’ motivation in reducing non-communicable diseases behavioral risk factors in the community.

**Methods:**

A randomized field trial study was conducted in 32 community health centers in 4 Iranian districts after a baseline population survey on the status of NCDs of 30–70-year-old individuals (*n* = 1225). The interventions were performed to improve insufficient physical activity, insufficient fruit consumption, insufficient vegetable consumption, high salt intake, and tobacco use. Four intervention packages were implemented in 24 community health centers; the other 8 centers were used as control groups. The non-physician community health workers performed the interventions. The packages additively included goal-setting, evidence-based education, operational planning, and incentive payments. A second survey was conducted 1 year after the start of the interventions to identify the effects on an independent random sample of 30–70-year-old individuals (*n* = 1221). Difference-in-difference method was used to quantify the interventions’ effects.

**Results:**

The average age of participants in both surveys was about 49 years. Also, about half of the participants were female, and about 43% were illiterate or had a primary school education. The interventions had statistically significant effects only on decreasing the prevalence of insufficient physical activity. The package with all the intervention components decreased the odds of insufficient physical activity to 0.24 (95% CI, 0.08, 0.72). The package with operational planning but no performance-based financing did not change the odds of insufficient physical activity.

**Conclusions:**

This study highlighted the importance of components, design, and implementation details of interventions intended to reduce NCDs behavioral risk factors. Some risk factors, such as insufficient physical activity, seem more easily modifiable with limited low-cost interventions in a one-year horizon. However, risk factors related to healthy food consumption and tobacco use need more extensive interventions.

**Trial registration:**

This trial was registered on the Iranian Registry of Clinical Trials (IRCT20081205001488N2) on 3 June 2018 (https://en.irct.ir/trial/774).

**Supplementary Information:**

The online version contains supplementary material available at 10.1186/s13690-023-01047-w.

## Background

Non-communicable diseases (NCDs) are the leading cause of death worldwide, as they accounted for 74% of total deaths and 85% of premature deaths in low- and middle-income countries (LMICs) in 2019 [1, 2]. In 2015, the United Nations (UN) acknowledged that the increase in the burden of NCDs is a major threat to its Sustainable Development Goals (SDGs) for the twenty-first century and targeted to reduce NDCs’ premature deaths to one-third by 2030 [3]. In Iran, NCD mortality increased from 49 to 82% from 1990 to 2017 [4]. In 2017, the loss of 7.0 million years of life was attributed to NDCs, indicating a 98% increase from 1990. Also, 15.0 million years were lived with disability due to NDCs, showing a 48% increase from 1990 [5, 6].

Structural social changes such as rapid urbanization, globalization, and population aging have accelerated the prevalence of NCDs [7]. Changes in social structures are usually followed by lifestyle changes and increased prevalence of behavioral risk factors such as unhealthy diet, physical inactivity, tobacco smoking, and alcohol use [8]. Behavioral risk factors precede the development of metabolic risk factors (e.g., raised blood pressure, overweight/obesity, and increased blood cholesterol) and can then further develop into NCDs [9]. According to the World Health Organization (WHO), 1.6 million preventable deaths per year are caused by physical inactivity, 7.2 million by tobacco use, 4.1 million by excess salt/sodium intake, and 3.9 million by inadequate fruit and vegetable consumption [10, 11]. These estimates indicate the importance of behavioral risk factors as preventable contributors to the development of NCDs [10].

In 2019, 16.5% of Iran’s deaths were attributed to an unhealthy diet, 14.1% to tobacco use, 4.4% to inadequate physical activity, and 1.2% to excess salt intake. The corresponding attributable DALYs to the risk factors were 7.5, 9.2, 1.9, and 0.6%, respectively [1]. Therefore, effective investments in curtailing NCDs risk factors can save and improve lives and will be economically justifiable if at-risk people are identified in the early stages [12].

Many cost-effective intervention methods to control NCDs have been introduced worldwide. However, the main challenge is implementing them in LMICs, which typically face a limited skilled workforce, financial resources, and community participation [13, 14]. Efforts to control the increasing rate of NCDs in Iran intensified in 2014 by launching an adaptation of the WHO’s Package of Essential, IraPEN [15]. However, the mismatch of typical training provided to health care workers with the extent of NCDs in the community, insufficient documentation to determine the existing NCDs’ status, inconsistency between training and practice, and instability of financial resources have obstructed the successful implementation of the package [16, 17].

In this study, a field trial was devised and carried out to address the above shortcomings. A set of intervention packages were designed to encourage reducing NCDs behavioral risk factors and implemented by non-physician community health workers (NPHWs) in a group of randomly selected community health centers (CHCs)—locally called Health Houses—in four Iranian districts. The NPHWs were informed about the status of NCDs behavioral risk factors in their catchment areas and encouraged to meet a set of goals to reduce the risk factors. Then, they randomly received a combination of different interventions: evidence-based training, an action plan to reduce the risk factors based on the NCDs status in their catchment areas, and incentive payments based on their performance in achieving the goals. The trial was conducted to understand the effects of these interventions on NCDs risk factors in the studied population.

## Methods

### Study sample

**Fig. 1:**
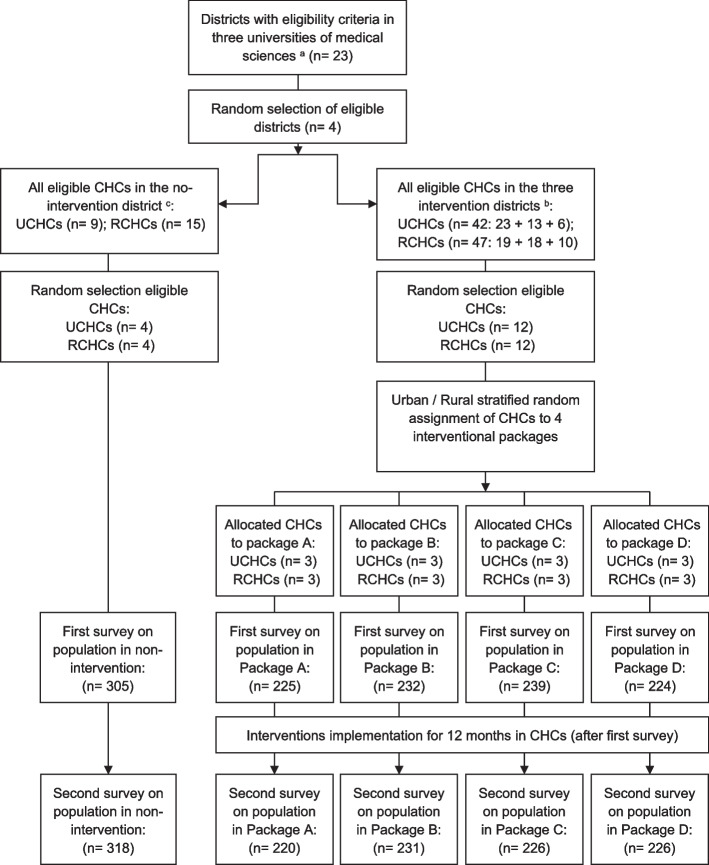
CONSORT flow diagram. Note: Analysed based on population in first and second surveys. CHCs, Community Health Centers; UCHCs, Urban Community Health Centers; RCHCs, Rural Community Health Centers. ^a^ These three universities are located in three provinces of Tehran, Semnan, and Bushehr in Iran. One of the universities’ health care systems’ key tasks is providing primary health services to the covered population. ^b^ Shahriar, Dashtestan, and Damghan. ^c^ Garmsar

Four districts (equivalent to counties) were selected for the field trial. One of the districts was the no-intervention district, and the other three were intervention districts. In each selected district, four urban and four rural CHCs were selected. In each of the 32 selected CHCs, a baseline survey was conducted on 30–70-year-old residents of its catchment area to understand the existing status of NCDs risk factors. The survey was administered from June to September 2018 using a Persian translation of an adapted WHO stepwise approach to surveillance (STEPS) questionnaires [18]. Then, four different intervention packages were randomly assigned to the selected urban or rural CHCs in the intervention districts. In any intervention district, one urban and one rural CHC received intervention package A, one urban and one rural CHC received intervention package B, and so on (Fig. 1). The intervention period was 12 months, after which the second survey was conducted on the same age population in the 32 CHCs to assess the impacts of the interventions from September to November 2019.

For each survey, a random sample of the population in the catchment area of each studied CHC was drawn. The sample size for each of the surveys was set to be 320 in each district; it was stratified by urban/rural CHCs, sex and age groups (30–39, 40–49, 50–59 and 60–69 years); 4 urban and 4 rural CHCs from each district were selected (totally, 12 urban and 12 rural in the intervention districts, in addition to the 4 urban and 4 rural in the “no intervention” district). So, the planned total sample size for each round of survey was 1280 [(12 + 12 + 4 + 4) * 40]. If we look at this number based on the intervention packages, the sample size was 240 for each of the intervention packages 1 to 4, and 320 for the no-intervention group [(4* 240) + 320 = 1280]. The sample size of 240 for each intervention group was enough to find a one-third decrease in the prevalence of 34% (based on a primary estimate for prevalence of physical inactivity) with an Alpha of 0.05 and a power of 80%. We used a higher sample size for the no-intervention group (320 versus 240) both to increase the power and to use a similar sampling protocole in all districts. As explained in the CONSORT flow diagram (Fig. 1), the final sample size in the 1st and 2nd surveys were 1226 and 1221 individuals, respectively. Since the cluster sample sizes were not proportional to the catchment areas’ population, sampling weights were used in all analyses. All interviews were in-person. If a household was not available at the first reach, it was contacted by the research team up to three times on three successive days to perform the interview. In each household, only one male and one female member from each of the following age group strata were interviewed: 30–39, 40–49, 50–59, and 60–70 years. If more than one male or female from an age group were living in a household, one of them was randomly selected and interviewed. A necessary inclusion criterion was informed consent by interviewees.

While the sampling method was similar in both surveys, the selected participants were not necessarily the same. Although the survey participants were expected to be among the target groups of the interventions, they were not necessarily the ones who received the intervention directly. According to the country’s Integrated Health Record System—called the SIB system locally [19]—on average, 20% of the catchment area population visit their local CHC in any given quarter. The country’s NPHWs’ reach, however, was expected to be much more than CHC’s direct utilization rate because, in addition to those who refer to them, they are responsible for improving the health of the entire population assigned to them. Based on how CHCs are structured and organized in the country, if individuals in a catchment area do not visit CHCs and demand health care, the NPHWs must reach out to them, encourage visits, or at least monitor their health remotely over the phone to make sure they are ceceiving health care somewhere else (such as the provate sector). This active follow-up method, especially in rural areas, has led to significant health improvements in the country, for example, remarkable decreases in maternal and childhood mortality and communicable diseases over several decades (Barzegar and Djazayeri 1981 [20], Rahbar and Ahmadi 2015 [21], and Keshvari et al. 2016 [22]). This study was built on the same infrastructures and intended to extend the NPHWs’ experiences regarding maternal and child health and communicable disease to non-communicable diseases.

Based on the approved protocol, the trial was planned to continue for 24 months, with a third survey at the end of the study. However, it was terminated prematurely after 12 months to comply with the country’s COVID-19 social distancing protocols.

The selected districts were Shahriar (population = 744,210), Dashtestan (population = 252,047), Damghan (population = 94,190), and Garmsar (population = 77,421) (Additional file 1). The districts’ populations are based on the country’s 2016 census [23]. A simple randomization method was used to select four urban and four rural CHCs in each district and to assign the intervention packages to the selected CHCs. Detailed explanations of the inclusion criteria for the districts, CHCs, and participants were explained in the protocol [24]. NPHWs implemented four intervention packages after receiving extensive training. Physicians were not the target group of this trial because they were undergoing a separate incentive payment scheme [25]. The CONSORT checklist can be found in Additional file 2 [26].

### The interventions and intervention packages

An intervention package in this study included the first, the first two, the first three, or all of the following interventions:

- *The first intervention (target-setting):* Short-term targets (e.g., decrease in tobacco use and salt consumption) were set based on the preliminary results obtained from the baseline survey and the national goals to control NCDs behavioral risk factors. The national goals were to reduce insufficient physical activity, insufficient fruit and vegetable, salt intake, and tobacco use by 20, 30, 30, and 30%, respectively, until 2025 [27, 28]. Specific quarterly and yearly targets for NCDs risk factors are reported in Additional file 3. Meetings were held with the NPHWs of the selected CHCs, and they were informed about the status of NCDs behavioral risk factors in their catchment area population and the national goals to reduce them. Also, the proposed targets were presented to them, and they were encouraged to work through achieving them. The research team did not go beyond providing information on the status of NCDs in the catchment areas and national NDC goals, setting the goals mentioned above, and encouraging the NPHWs to achieve them. *The second intervention (evidence-based education):* The research team set up a 16-hour workshop for the NPHWs’ of the CHCs that received this intervention. The workshops were merely informational, during which the adverse health effects of overconsumption of salt, underconsumption of fruit and vegetable, insufficient physical activity, and tobacco use were extensively discussed. The trainees were provided with the related informational brochures as well. In addition, the team used Disease Control Priorities, 3rd edition [28, 29], and the Iranian version of the WHO package of essential NCDs (PEN) [30] to prepare a review summary of the effectiveness of the interventions aimed at decreasing the risk of NCDs in LMICs. The review also included success stories in other countries and methods of selecting, planning, and implementing cost-effective interventions. For the review.
- *The third intervention (operational planning):* The research team coordinated with NPHWs and the local health experts to collaboratively devise operational plans for the selected CHCs in a 12-hour workshop. The major component of the NPHWs’ action plans was periodic (biweekly or monthly) educational sessions for the covered population on the causes and detrimental health effects of NDCs and practical methods to decrease the risk of NDCs (such as increasing physical activities, adjusting the diet, reading food products’ nutrition label, decreasing the consumption of salt, canned and fast food, and the use of tobacco products). Healthy lifestyle (especially in regards to movement and diet) and smoking reduction counseling was also offered to the CHC visitors. Other action plan items were organizing weekly public walking events, setting up group activities (such as painting, reading, and board games), and coordinating with government-owned sports facilities to provide free hours to the public (specifically, three two-hour sessions a week). The focus of the action plan was different from one CHC to another based on the finding of the baseline survey at the CHC’s catchment area. For example, if the body mass index was particularly high in a catchment area, more frequent educational sessions were set on the risk of obesity and the importance of physical activity, and more public walking events were organized by the NPHWs.
  To support the action plans’ execution, the team allocated a supportive budget for the devised operational plans. The maximum supportive budget was 60 million Rials—equivalent to 556 United States dollars, USD, based on the exchange rate of 107,832 Rial/USD at the time of study [31]. The budget could be spent on purchasing equipment for the CHC (for example, digital blood pressure sphygmomanometers, body weight scales, and height measuring devices) and materials for educational sessions, group sports, and non-sport events.
- *The fourth intervention (performance-based financing or PBF):* NPHWs of the selected CHCs received incentive payments. The payments were calculated at the CHC level, and paid to all NPHWs of that CHC per the pre-defined targets every 3 months. The average level of achievement of each center to its 3-month targets for eight different NCD behavioral and metabolic risk factors was quantified. The CHCs were then classified based on the percentage of their achievements into one of the following four groups: < 25, 25–49.99, 50–62.49, and 62.5% or more. These groups, respectively, received no incentive, one-third, two-thirds, and full incentive. The full monthly incentive was 10% of the average monthly salary of a typical NPHW in the studied districts, which was determined to be approximately 25 million Rial (or 232 USD). Therefore, the maximum monthly incentive payment was approximately 23 USD. No payment was delayed because they were made directly to the NPHWs’ bank account immediately after each assessment.

Intervention package A included only the first intervention, goal setting. Intervention package B included the first two interventions: goal setting and evidence-based training. Intervention package C included the first two interventions plus an action plan. In intervention package D, PBF was added to other interventions (Table 1). CHCs that received the intervention packages A, B, C, and D were also called intervention groups A, B, C, and D, respectively, in this study. The no-intervention district (Garmsar district) received neither of the interventions.

**Table 1:** Assignment of interventions to CHCs inside each of the three treatment districts

Intervention Package	Urban/Rural Separation	Intervention:			
		Target-Setting	Evidence-Based Education	Operational Planning	Performance-Based Financing
A	1 Rural, 1 Urban	Yes	No	No	No
B	1 Rural, 1 Urban	Yes	Yes	No	No
C	1 Rural, 1 Urban	Yes	Yes	Yes	No
D	1 Rural, 1 Urban	Yes	Yes	Yes	Yes

Every two to four weeks, the implementation status of the interventions was reviewed and checked by the district and province supervisors selected by the research team. Also, reports on the interventions were received by the research team every quarter. The reports contained performance reviews per the set goals. Moreover, they included detailed accounts of the activities conducted at the CHCs that received an action plan. The key components of the reports were activities’ dates and type of the activity and the number of participants.

### Statistical analysis

This study’s objective was to compare NCDs behavioral risk factors before and after the interventions and identify effective interventions. NCDs behavioral risk factors analyzed in this study were zero-one indicators of insufficient physical activity, insufficient fruit consumption, insufficient vegetable consumption, high salt intake, and tobacco use.

Not meeting the WHO recommendations on physical activity (Metabolic Equivalent of Task, MET, less than 600 METs per week) was defined as insufficient physical activity [32]. The WHO’s recommendations were used to determine insufficient fruit (less than two medium-sized fruits, such as two medium apples or half a cup of nuts, in the last 24 hours) and vegetable consumption (less than three cups of raw leafy vegetables or one and a half cups of cooked or chopped vegetables in the last 24 hours) [33–35]. A person was identified as a high salt consumer if the person always or often added salt or salt additives to the food [36]. Current tobacco smoking was defined as the use of any tobacco products, including cigarettes, cigars, or pipes, on a daily or non-daily basis in the last 30 days [37].

The prevalence of each NCDs risk factor in the baseline and second surveys was calculated in populations assigned with each intervention package. Then, the difference in the prevalence rates between the two surveys was calculated. For the more formal analysis of the effect of the designed intervention packages, the difference-in-difference (DID) design was employed. The following equation shows the linear specification of the DID design:

1 $${Y}_{ic t}=\alpha +\beta {IntPackage}_{ic}+\gamma {Post}_{it}+\rho \left({IntPackage}_{ic}\times {Post}_{it}\right)+\theta {CHC}_{it}+\delta {X}_{ic t}+{\varepsilon}_{ic t}$$

where *i* indicates a surveyed individual, *c* indicates the community health center to which the individual is affiliated, and *t* indicates the survey year. The dependent variable, *Y*, is a binary variable that indicates one of the NCDs behavioral risk factors for the individual. The variable *IntPackage* is a categorical variable with five values (0, 1, 2, 3, and 4), indicating the intervention package assigned to the CHC where the individual receives health services (Table 1). The value 0 was assigned to individuals surveyed in the no-intervention district. Therefore, four *β* s were estimated. Estimations of *β*_*A*_, *β*_*B*_, *β*_*C*_, and *β*_*D*_ provide an adjusted comparison of the average level of dependent variable at the catchment areas that received intervention packages A, B, C, and D, respectively, to that in the non-intervention district. The variable *Post* indicates the survey year. It takes the value 0 if the individual was surveyed before the implementation of the interventions (2018) and 1 if surveyed after the trial (2019). The variable *CHC* is a community health center indicator (1, 2, …, 32), as any surveyed individual is affiliated with a specific CHC. This variable accounts for the influence of all unobservable/unmeasurable CHC-specific confounders that might not change over the study period (e.g., health care resources in the community, attitudes towards using modern medicine versus traditional practices, average distance from the CHCs, and overall weather patterns). The variable *X* is a vector of socioeconomic factors including age, sex, marital status (in three categories: never married, married, divorced or widowed), the level of education (in four categories: illiterate or primary, secondary, high school, and some college), labor market status (in six categories: public wage and salary job, private wage and salary job, self-employed, homemaker, retired, and unemployed), health insurance status, and homeownership status. The coefficients of interest in this specification are *ρ*s (i.e., *ρ*_*A*_, *ρ*_*B*_, *ρ*_*C*_, and *ρ*_*D*_) which show the effect of the intervention packages versus no intervention among those surveyed after the intervention.

Given the binary nature of the outcome variables in this study, logistic models were used in fitting Eq. 1. Odds ratios were calculated, representing the change in the odds of the dependent variable being equal to 1 due to one unit change in either of the terms on the right-hand side of Eq. 1. Equation 2 is the logistic transformation of Eq. 1.

2 $$\mathit{\ln}\left(\frac{p_{Y_{ic t}}}{1-{p}_{Y_{ic t}}}\right)=\alpha +\beta {IntPackage}_{ic}+\gamma {Post}_{it}+\rho \left({IntPackage}_{ic}\times {Post}_{it}\right)+\theta {CHC}_{it}+\delta {X}_{ic t}+{\epsilon}_{ic t}$$

where ${p}_{Y_{ict}}$ is the probability of the dependent variable *Y*_*ict*_ being reported as 1.

To account for the possibility that the NCDs behavioral risk factors (the *Y*s) may not be independently distributed within the population covered by each community health center, hence the estimated standard error be artificially low, standard errors were clustered at the CHC level [38]. Also, a sampling weight was assigned to the surveyed individuals. Sampling weights were calculated as the inverse of the ratio of sampled individuals characterized by sex and age group relative to the number of individuals in the respective sex and age group (namely, 30–39, 40–49, 50–59, and 60–70) in the population. For a specific individual, the ratio was the multiplication of two shares: (1) the share of surveyed individuals of the same sex and age group in the corresponding catchment area’s population, and (2) the share of individuals of the same sex and age group in the corresponding district in the country’s population. The shares were separately calculated for urban and rural areas.

The estimations were done with and without adjusting for the socioeconomic factors (*X*) to assess the extent of any potential observable bias in the selection of CHCs and the assignment of intervention packages. The statistical package used for analyses was STATA 14.0 (Stata, Inc., College Station, Texas).

### Ethical issues/statement

This study has been approved by the national committee on ethics in medical research (code: IR.NIMAD.REC.1396.084) as well as our institutional review board (code: IR.IUMS.REC.1395.1057613). Written informed consent has been obtained from study participants.

## Results

A total of 2446 people aged 30–70 years participated in the two surveys, 1225 individuals in the first and 1221 in the second survey. The socioeconomic characteristics of survey participants in both rounds were strikingly similar. For example, the mean age and sex ratio of the participants in the two surveys were very similar (about 49 years and about 50% women). Approximately 43% had illiterate or primary school education, while about 11% had a college education. About 5% were employed, 24–27% were self-employed, 93–95% were insured, and 84–85% were homeowners (Table 2).

**Table 2:** Demographic and economic characteristics of participants in each group in the two surveys

Socioeconomic Factors	Total *n* = 2446		Intervention Package/Group									
			A *n* = 445		B *n* = 463		C *n* = 465		D *n* = 450		None *n* = 623	
	Survey 1 *n* = 1225	Survey 2 *n* = 1221	Survey 1 *n* = 225	Survey 2 *n* = 220	Survey 1 *n* = 232	Survey 2 *n* = 231	Survey 1 *n* = 239	Survey 2 *n* = 226	Survey 1 *n* = 224	Survey 2 *n* = 226	Survey1 *n* = 305	Survey 2 *n* = 318
Mean Age (Standard Deviation)	49.3 (0.33)	49.4 (0.32)	49.5 (0.76)	49.6 (0.74)	49.3 (0.73)	49.3 (0.76)	49.9 (0.73)	49.1 (0.74)	48.9 (0.78)	49.1 (0.73)	48.8 (0.65)	49.7 (0.63)
***p*-value**	0.831	0.968										
Sex:												
Male (%)	611 (49.9)	606 (49.6)	112 (49.8)	107 (48.6)	117 (50.4)	117 (50.6)	113 (47.3)	107 (47.4)	110 (49.1)	117 (51.8)	159 (52.1)	158 (49.7)
Female (%)	614 (50.1)	615 (50.4)	113 (50.2)	113 (51.4)	115 (49.6)	114 (49.4)	126 (52.7)	119 (52.6)	114 (50.9)	109 (48.2)	146 (47.9)	160 (50.3)
***p*-value**	0.853	0.899										
Education:												
Illiterate or Primary School (%)	497 (43.5)	517 (42.5)	105 (50.0)	122 (55.5)	109 (49.1)	116 (50.4)	117 (52.5)	121 (53.8)	88 (47.3)	83 (36.7)	78 (25.8)	75 (23.7)
Secondary School (%)	158 (13.8)	196 (16.1)	19 (9.0)	35 (15.9)	39 (17.6)	39 (17.0)	20 (9.0)	30 (13.3)	27 (14.5)	44 (19.5)	53 (17.6)	48 (15.1)
High School (%)	365 (31.9)	368 (30.2)	65 (31.0)	43 (19.6)	58 (26.1)	64 (27.8)	68 (30.5)	52 (23.1)	45 (24.2)	70 (31.0)	129 (42.7)	139 (43.9)
Some College (%)	123 (10.8)	137 (11.2)	21 (10.0)	2 (9.0)	16 (7.2)	11 (4.8)	18 (8.0)	22 (9.8)	26 (14.0)	29 (12.8)	42 (13.9)	55 (17.3)
***p*-value**	0.001>	0.001>										
Marital Status:												
Never Married (%)	82 (7.0)	84 (6.9)	12 (5.7)	7 (3.2)	9 (4.0)	13 (5.7)	18 (8.1)	12 (5.4)	19 (9.0)	22 (9.7)	24 (7.9)	30 (9.4)
Married (%)	996 (85.4)	1039 (85.4)	187 (89.5)	198 (90.4)	190 (86.0)	194 (84.7)	187 (84.2)	198 (88.4)	176 (83.0)	185 (81.9)	256 (84.8)	264 (83.0)
Divorced/Widowed (%)	88 (7.6)	93 (7.7)	10 (4.8)	14 (6.4)	22 (10.0)	22 (9.6)	17 (7.7)	14 (6.2)	17 (8.0)	19 (8.4)	22 (7.3)	24 (7.6)
***p*-value**	0.456	0.581										
Job:												
Public Wage and Salary (%)	101 (8.7)	75 (6.2)	13 (6.3)	8 (3.6)	18 (8.1)	14 (6.1)	16 (7.2)	15 (6.8)	20 (9.4)	13 (5.8)	34 (11.4)	25 (7.9)
Private Wage and Salary (%)	104 (9.0)	92 (7.6)	21 (10.1)	31 (14.0)	21 (9.5)	15 (6.5)	28 (12.7)	21 (9.6)	12 (5.7)	18 (8.1)	22 (7.4)	7 (2.2)
Self-Employed (%)	316 (27.2)	292 (24.1)	53 (25.5)	46 (20.8)	54 (24.3)	56 (24.6)	32 (14.5)	49 (22.3)	86 (40.6)	58 (26.0)	91 (30.5)	83 (26.1)
Homemaker (%)	491 (42.3)	563 (46.5)	89 (42.8)	103 (47.1)	98 (44.1)	110 (47.8)	110 (49.8)	105 (47.7)	73 (34.4)	102 (45.7)	121 (40.6)	143 (45.0)
Retired (%)	91 (7.8)	132 (10.9)	20 (9.6)	24 (10.9)	19 (8.6)	24 (10.4)	22 (10.0)	19 (8.6)	12 (5.7)	25 (11.2)	18 (6.0)	40 (12.6)
Unemployed (%)	58 (5.0)	57 (4.7)	12 (5.8)	8 (3.6)	12 (5.4)	11 (4.8)	13 (5.9)	11 (5.0)	9 (4.25)	7 (3.1)	12 (4.0)	20 (6.2)
***p*-value**	0.031	0.307										
Health Insurance:												
Insured (%)	1035 (92.6)	1152 (94.7)	203 (96.2)	209 (95.4)	209 (94.6)	205 (90.3)	171 (96.1)	210 (92.9)	164 (78.8)	215 (95.1)	288 (96.0)	313 (98.4)
Uninsured (%)	83 (7.4)	64 (5.3)	8 (3.8)	10 (4.6)	12 (5.4)	22 (9.7)	7 (3.9)	16 (7.1)	44 (21.2)	11 (4.9)	12 (4.0)	5 (1.6)
***p*-value**	0.001>	0.001										
Homeownership:												
Yes (%)	898 (83.8)	1027 (85.1)	183 (87.6)	171 (79.2)	170 (86.3)	209 (92.9)	164 (92.7)	192 (86.1)	135 (70.7)	175 (77.4)	246 (82.8)	280 (88.3)
No (%)	173 (16.2)	180 (14.9)	26 (12.4)	45 (20.8)	27 (13.7)	16 (7.1)	13 (7.3)	31 (13.9)	56 (29.3)	51 (22.6)	51 (17.2)	37 (11.7)
***p*-value**	0.001>	0.001>										

There was no significant difference in the average age and sex of survey participants from catchment areas of no-intervention and intervention districts. However, the share of participants with primary or no education in the non-intervention district (25.8 and 23.7% in the first and second surveys) was much lower than that in the intervention districts (between 36.5 and 55.5%). Consequently, there were more participants with a high school or a college education in the non-intervention than in intervention districts. In addition, the share of high school-educated participants who were potentially exposed to intervention package A decreased from 31.0% in survey 1 to 19.6% in survey 2, an 11.4% decrease; the share of illiterate or primary school-educated participants who were potentially exposed to intervention package D decreased from 47.3% in survey 1 to 36.7% in survey 2, an 11.6% decrease. Most surveyed individuals were married, regardless of the intervention packages assigned to their pertinent CHCs: the share of married participants was between 81.9 and 90.4% across intervention groups and surveys. Also, the majority of participants were either homemakers (between 34.4 and 49.8%) or self-employed (between 14.5 and 40.6%), had health insurance (between 78.8 and 98.4%), and were homeowners (between 70.7 and 92.9%) (Table 2). Given the observed variations in the characteristics, they were used in adjusting the estimated effects of the intervention packages.

The crude comparison of the levels of NCDs behavioral risk factors in each intervention group before and after the interventions showed the largest decrease in insufficient physical activity in intervention group D with a 29% decrease (95% Confidence Interval: 20%, 38%), then in intervention groups B and A with 25% (95% CI: 17%, 34%) and 15% (95% CI: 6%, 24%) decreases, respectively. The observed level of physical activity did not change in the control group during the study period.

A large and consistent increase in fruit consumption was also observed in all intervention and non-intervention groups. We suspect the increase in fruit consumption was largely attributable to the strikingly lower relative prices for fruits during the study period (Additional file 4). No consistent and statistically significant pattern of increase or decrease in fruit and vegetable consumption, high salt intake, and tobacco use was observed in the studied groups (Table 3).

**Table 3:** The difference in NCDs behavioral risk factors between the two surveys

NCDs Risk Factors	Intervention Package ^a^ / Group	First Survey (%)	Second Survey (%)	Difference (%) (95% Confidence Interval)
Insufficient Physical Activity	A	40	25	−15 (−24, −6)
	B	49	24	−25 (−34, −17)
	C	41	40	−1 (−11, 8)
	D	53	24	−29 (−38, −20)
	None	39	40	1 (−7, 8)
Insufficient Fruit Consumption	A	66	57	−9 (−19, 0)
	B	59	53	−6 (−1, 3)
	C	68	51	−17 (−27, −8)
	D	72	59	−13 (−22, −4)
	None	73	56	−17 (−25, −10)
Insufficient Vegetable Consumption	A	32	34	2 (−6, 11)
	B	33	39	6 (− 2, 15)
	C	37	36	− 1 (− 11, 9)
	D	40	47	7 (− 2, 16)
	None	38	37	− 1 (−9, 7)
High Salt Intake	A	15	13	−2 (−9, 5)
	B	19	22	3 (−4, 11)
	C	5	15	10 (4, 16)
	D	19	22	3 (−5, 11)
	None	15	13	−2 (− 8, 3)
Current Tobacco Use	A	16	20	4 (− 3, 11)
	B	20	19	− 1 (− 8, 7)
	C	16	22	6 (− 2, 12)
	D	14	14	0.3 (− 6, 7)
	None	18	11	−7 (− 13, 1)

*NCDs* Non-communicable diseases

^a^ Intervention package A included target-setting. Intervention package B included A plus evidence-based education. Intervention package C included B plus operational planning. Intervention package D included plus performance-based financing

The results of the statistical analyses largely confirmed the results of the crude comparisons. Using the non-intervention district as the reference group and employing a DID research design (Eq. 2), no improvement in fruit and vegetable consumption, high salt intake, and tobacco use was estimated. However, fairly consistent improvements in the level of physical activity in all intervention groups were observed compared to the non-intervention group. Among the intervention packages, B and D resulted in decreasing insufficient physical activity. Specifically, the unadjusted estimations showed that the odds of reporting insufficient physical activity decreased to 0.32 (95% CI: 0.11, 0.88) among the surveyed individuals covered by CHCs that received the intervention package B, and to 0.28 (95% CI: 0.10, 0.75) among those covered by CHCs that received the intervention package D. These results were confirmed after adjusting for socioeconomic factors: 0.27 (95% CI: 0.09, 0.85) decrease in the odds of insufficient physical activity in intervention group B, 0.24 (95% CI: 0.08, 0.72) in intervention group D (Table 4). In other words, the package with all intervention components (which added the provision of an action plan and incentive payments to the previous two) decreased the likelihood of insufficient physical activity by 76% (95% CI: 28%, 92%).

**Table 4:** The estimated effects of intervention packages on the NCDs behavioral risk factors

NCDs Risk Factor	Intervention Package ^a^	Unadjusted		Adjusted for Socioeconomic Factors	
		Odds Ratio (95% CI)	*p*-value	Odds Ratio (95% CI)	*p*-value
Insufficient Physical Activity	A	0.49 (0.20, 1.22)	0.12	0.56 (0.21, 1.51)	0.25
	B	0.32 (0.11, 0.88)	0.02	0.27 (0.09, 0.85)	**0.02**
	C	0.91 (0.25, 3.40)	0.89	0.81 (0.17, 3.76)	0.78
	D	0.28 (0.10, 0.75)	0.01	0.24 (0.08, 0.72)	**0.01**
	None	Reference Group			
Insufficient Fruit Consumption	A	1.44 (0.35, 5.95)	0.61	1.52 (0.27, 8.62)	0.63
	B	1.70 (0.45, 6.35)	0.43	1.94 (0.38, 9.84)	0.42
	C	1.04 (0.23, 4.72)	0.95	0.99 (0.13, 7.32)	0.99
	D	1.19 (0.35, 4.03)	0.78	1.42 (0.31, 6.61)	0.64
	None	Reference Group			
Insufficient Vegetable Consumption	A	1.17 (0.37, 3.71)	0.79	1.13 (0.26, 4.89)	0.86
	B	1.39 (0.53, 3.62)	0.51	1.71 (0.64, 4.54)	0.28
	C	1.00 (0.34, 2.91)	0.99	0.70 (0.19, 2.62)	0.59
	D	1.39 (0.44, 4.42)	0.57	1.71 (0.46, 6.43)	0.42
	None	Reference Group			
High Salt Intake	A	1.01 (0.33, 3.11)	0.98	1.04 (0.32, 3.38)	0.94
	B	1.45 (0.51, 4.12)	0.48	1.23 (0.41, 3.70)	0.71
	C	3.92 (1.05, 14.63)	0.04	4.35 (0.80, 23.47)	0.08
	D	1.43 (0.37, 5.56)	0.61	1.38 (0.36, 5.33)	0.64
	None	Reference Group			
Current Tobacco Use	A	2.37 (1.09, 5.18)	0.03	1.87 (0.83, 4.23)	0.13
	B	1.73 (0.65, 4.59)	0.27	1.47 (0.50, 4.29)	0.48
	C	2.54 (0.84, 7.66)	0.09	1.55 (0.56, 4.29)	0.40
	D	1.82 (0.81, 4.12)	0.15	1.24 (0.44, 3.54)	0.68
	None	Reference Group			

Boldface indicates statistical significance (*p* < 0.05)

*NCDs* Non-communicable diseases

^a^ Intervention package A included target-setting. Intervention package B included A plus evidence-based education. Intervention package C included B plus operational planning. Intervention package D included plus performance-based financing

## Discussion

The frontline CHCs in rural areas were piloted in the 1970s and expanded rapidly to a nationwide network in the early 1980s. Each CHC has at least one female and one male community health worker. Most of them are NPHWs, usually selected from local people and trained in a specific practical program designed by the country’s Ministry of Health. Their focus has been providing primary health care services, including health education, promotion of food supply and proper nutrition, advocacy for and monitoring of water safety and basic sanitation, maternal and child health, immunization according to the national immunization program, prevention and control of locally important diseases, treatment of common diseases and injuries, and provision of essential medications [20]. In the past two decades, they have been more involved in managing NCDs risk factors, and there is indirect evidence of the success of their role in this area [39]). The functions of the urban CHCs are close to their rural counterparts. Each urban CHC has at least one NPHW for every 2500 individuals in the catchment area. The average coverage of rural and urban CHCs is currently 1200 and 12,500 individuals, respectively. In either case, urban or rural, people covered by a CHC cannot use the services of other CHCs. In rural areas, especially in remote areas, utilization of CHC services is usually high. In urban areas and especially in large cities, there are several alternatives, usually more specialized, in public and private sectors for all kinds of health services but with a higher out-of-pocket cost. Also, active follow-up of the target population for health services in urban centers is not as usual and orderly as in rural centers [40].

This trial used the aforementioned community health foundation to examine the effectiveness of four interventional packages designed to improve four major NCDs behavioral risk factors, namely, insufficient physical activity, insufficient fruit and vegetable consumption, high salt consumption, and current tobacco use. NPHWs implemented the interventions at a randomly selected number of CHCs in four Iranian districts. The most basic intervention package included target-setting for NPHWs. Evidence-based education, operational planning, and PBF for NPHWs were added to target-setting in other packages. Improvements were observed in only one NCDs behavioral risk factor, insufficient physical activity. The most effective intervention package included all four interventions. In this study, incentives were paid to enhance the achievement of public health goals. The finding is in line with the result of previous studies that have shown increasing the motivation of NPHWs can pave the way for achieving predetermined goals in areas where access to physicians is limited [41, 42].

Our findings accord with some review studies which shown that interventions in primary health care can significantly improve physical activity compared to other NCDs behavioral risk factors, even 6 to 12 months after intervention [43, 44]. A randomized controlled trial (RCT) in the United Kingdom showed that motivational interviewing of primary care patients by NPHWs improved physical activity levels assessed 12 months after the intervention [45]. Other RCTs also showed interventions with multiple lifestyle improvement components can increase physical activity [46–48].

This study’s measured improvements in physical activity can be attributed to the provision of regular physical activity programs by NPHWs to the CHC visitors, NPHWs’ active involvement in physical activity initiatives, and encouraging local councils to waive recreational areas’ and sports facilities’ usage fees for group activities supported by CHCs. The finding that the addition of PBF to other interventions made the results stronger indicates the importance of incentive payments and their role in promoting NPHWs’ efforts. A similar study in San Diego, California, USA, included direct involvement of health workers in public exercises and group walking over 12 months and measured a noticeable increase in physical activity in the community [49, 50]. Other studies have also shown the effectiveness of community-based physical activity interventions [51, 52].

An irregularity was found in the results for physical activity. Adding more components to the intervention package was expected to make it more effective. Accordingly, adding evidence-based training to goal-setting made the effect larger and statistically significant. However, adding an action plan to the intervention package without PBF eradicated the effect of goal-setting and evidence-based training. Adding PBF to the package that included goal-setting, evidence-based training, and an action plan, on the other hand, significantly increased the magnitude of the effect. Therefore, an action plan without incentive payment appeared to be ineffective. This irregularity may be an important result of this study. Anecdotal evidence collected by the authors showed that some NPHWs of CHCs who received the intervention package with and without incentive payments (packages C and D) might be communicating during the study period, although the research team set up workshops for different interventions at different weeks and advised district health care authorities to keep the intervention assignments to the CHCs in their district confidential. Hence, no incentive payment to NPHWs who implemented intervention package C might have acted as a disincentive, perhaps because they were expected to do the same amount of work as NPHWs who implemented intervention package D.

Comparing the effects on physical activity of intervention packages B and D, we found no discernable difference: the adjusted odds ratios for intervention packages B and D were 0.27 (95% CI: 0.09, 0.85) and 0.24 (95% CI: 0.08, 0.72), respectively. This is another unexpected result, indicating that a package with embedded financial incentives (D) did not improve the results over a package without the incentives (B). This finding highlights the importance of providing evidence-based training to the participating NPHWs.

The goal of increasing fruit and vegetable consumption may be achievable by spending more time and holding counseling sessions, as other studies have shown [53, 54]. On the other hand, failure to achieve this goal might be due to the high inflation rate in the country during the study period, especially the sharp increase in food, fruit, and vegetable prices because of a new round of international economic sanctions on the country from 2018 [55]. Virtually, the average cost of food in 2019 was estimated to be 3.6 times more than that in 2017 [56]. Factors influencing failure to achieve the desired effect of interventions in the consumption of fruit and vegetable have been reported before. Poor nutritional knowledge [57], the role of media in advertising food with low nutritional value [58], and the high relative price of fruits [59] are posed as the main factors.

Measuring no improvement in reducing high salt intake in this study is consistent with the contemporary consumption habits in the country and the challenges of changing them. For example, a WHO report indicated that in 2012, salt consumption in Iran was twice the recommended level [60, 61]. Evidence has shown that changing behavior and modifying consumption patterns require comprehensive and extensive programs [62]. Other studies have shown that changing the habit of salt consumption is less probable only with educational and information campaigns [63, 64]. One potential challenge in reducing salt intake in rural Iran may be the presence of traditional healers who disseminate rumors mixed with religious stories about the benefits of using salt, salt stone, and sea salt, even for people with hypertension [65].

No measured effect of this study’s interventions on tobacco use was expected as influencing tobacco use habits faces easy access and low price challenges. The smoking economics literature shows that policies that increase the price of tobacco products (through increased sales taxes, for example) are most influential in decreasing tobacco use [66–70]. Such macro-level policies were out of this study’s scope.

This study has several limitations. First, only short-term effects were measured because individuals in the intervention CHCs were not followed up. The trial was initially designed for 24 months [24]. Nonetheless, in compliance with limitations imposed by the emergence of the COVID-19 pandemic and the required social distancing policies, the study was stopped prematurely. Similar studies with longer periods of intervention might be necessary to measure longer-term effects on NCDs behavioral risk factors. Second, the survey participants were not necessarily the ones who were directly affected by the interventions. Although the influence of a typical CHC’s activities in the country is expected to go beyond direct visits, the measured effects may only reflect the lower bounds of the actual effects on the treated individuals. Third, participating NPHWs’ relocation to serve in other CHCs was out of the researchers’ control. Nonetheless, no relocation took place between the selected intervention CHCs. The research team traced all relocations monthly and provided the replacing NPHWs with the same training. Fourth, implementing simultaneous national research projects, such as the High Blood Pressure Campaign, was not under the researchers’ control [71]. However, such factors are expected to affect all intervention and non-intervention groups similarly. The fifth is a general limitation of studies that target NPHWs. Although we used training workshops to mprove NPHWs’ knowledge about non-communicable diseases, and developing their skills in applying the trial’s designed interventions, we do not believe these efforts would replace formal academic or occupational training or employing more skilled health workers. This might be one of the reasons for not measuring an effect on most behavioral risk factors in this study.

## Conclusion

Paying incentives to NPHWs, along with other interventions, could be considered a useful means of improving physical activity in the community. This study could not be continued because of COVID-19. Longer studies are needed to identify the long-term effects of such interventions.

## Supplementary Information

**Additional file 1.** Districts of intervention and no intervention in IRPONT study.

**Additional file 2.** CONSORT checklist.

**Additional file 3.** Specific quarterly and yearly targets for NCDs risk factors.

**Additional file 4. **Supplementary analysis of fruits/ vegetables consumption

**Additional file 5.** IRPONT collaborators.

## Data Availability

The datasets generated and/or analyzed during the current study are not publicly available as publications are planned but are available from the corresponding author upon reasonable request.

## Abbreviations

NCDs: Non-communicable diseases
UN: United Nations
LMICs: Low- and Middle-Income Countries
SDGs: Sustainable Development Goals
WHO: World Health Organization
IraPEN: Iranian Package of Essential Non-communicable diseases
NPHWs: Non-Physician community Health Workers
CHCs: Community Health Centers
STEPS: Stepwise approach to Surveillance
COVID-19: Coronavirus Disease of 2019
PBF: Performance-Based Financing
MOHME: Ministry of Health & Medical Education
MET: Metabolic Equivalent of Task
DID: Difference-In-Difference
RCT: Randomized Controlled Trial
