# Supplementary material for: Motivating non-physician health workers to reduce the behavioral risk factors of non-communicable diseases in the community: a field trial study
Source: Arch Public Health. 2023 Mar 10;81:37. doi: 10.1186/s13690-023-01047-w (PMC9998263; doi:10.1186/s13690-023-01047-w)
Supplement: Supplementary file 1 — Additional file 1. Districts of intervention and no intervention in IRPONT study. [file 13690_2023_1047_MOESM1_ESM.docx]

Additional file 1. Districts of intervention and no intervention in IRPONT study


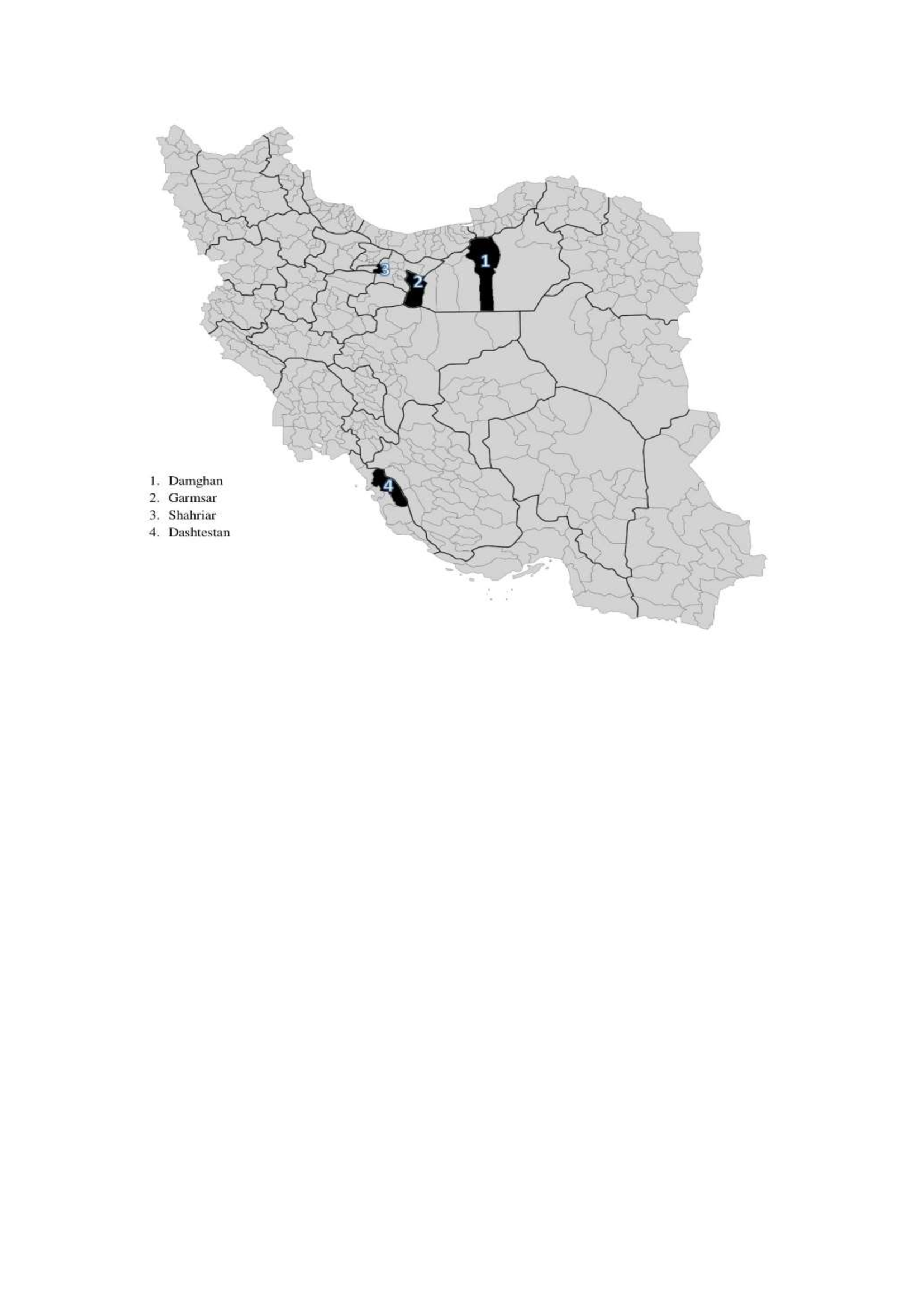


Source: This is an edited version of the map downloaded from <https://en.wikipedia.org/wiki/List_of_cities_in_Iran_by_province#/media/File:Iran_Counties_by_Population_(2021).svg>
