# Supplementary material for: Motivating non-physician health workers to reduce the behavioral risk factors of non-communicable diseases in the community: a field trial study
Source: Arch Public Health. 2023 Mar 10;81:37. doi: 10.1186/s13690-023-01047-w (PMC9998263; doi:10.1186/s13690-023-01047-w)
Supplement: Supplementary file 3 — Additional file 3. Specific quarterly and yearly targets for NCDs risk factors. [file 13690_2023_1047_MOESM3_ESM.docx]

Additional Table. Specific quarterly and yearly targets for NCDs risk factors

|  | The Targeted Reduction | |
| --- | --- | --- |
| NCDs Behavioral Risk Factor | Per Quarter | Per Year |
| Insufficient Physical Activity | 0.41%−1.25% | 1.64%−5.00% |
| Insufficient Fruit & Vegetable Intake | 1.25%−5.00% | 5.00%−15.00% |
| Salt Intake | 0.83%−2.50% | 3.32%−10.00% |
| Tobacco Use | 0.41%−1.25% | 1.64%−5.00% |
| NCDs Metabolic Risk Factor |  |  |
| Hypertension | 0.63%-1.90% | 2.52%-7.60% |
| Hyperlipidaemia | 1.25%-3.75% | 5%-15% |
| Obesity & overweight | Stop | Stop |
| Diabetes | Stop | Stop |
