# Supplementary material for: Motivating non-physician health workers to reduce the behavioral risk factors of non-communicable diseases in the community: a field trial study
Source: Arch Public Health. 2023 Mar 10;81:37. doi: 10.1186/s13690-023-01047-w (PMC9998263; doi:10.1186/s13690-023-01047-w)
Supplement: Supplementary file 4 — Additional file 4. Supplementary analysis of fruits/ vegetables consumption [file 13690_2023_1047_MOESM4_ESM.docx]

Additional File 4. Supplementary analysis of fruits/ vegetables consumption

In general, the percentage of surveyed individuals who reported insufficient fruit consumption decreased from the first to the second survey. The decrease was larger among those living in the catchment area of the CHCs receiving intervention package C (17%), intervention package D (13%), and no intervention (17%). We suspect the decreases were largely attributable to the relatively lower fruit prices during the study period.

The second survey was conducted during the months 6 to 9 on the Persian calendar. These months overlap with September to December on the western/Gregorian calendar. In the following table, we have provided the inflation rate (percentage change in consumer price index, CPI) for the 12 months period leading to each second-survey month for the provinces where the trial was performed. The rates are calculated and provided by the Central Bank of Iran at LNIK. No district-level price index and inflation are available

Food and beverage inflation was greater than the overall inflation in all months and provinces. Strikingly, however, fruit and nuts inflation (fruits and nuts are not separated in the Iranian CPI data) was the distant lowest inflation among all food items in all four months overall in the country and the three trial provinces. In the non-intervention province, fruit and nuts inflation was lower than in the other provinces.

| **Percentage change in the consumer price index (inflation) with respect to the same month in the previous year in each second-survey month** | | | | |
| --- | --- | --- | --- | --- |
| Persian Months: → | 6: Shahrivar | 7: Mehr | 8: Aabaan | 9: Aazar |
| Gregorian Month: → | Aug-Sep | Sep-Oct | Oct-Nov | Nov-Dec |
|  | County |  |  |  |
| All Goods & Services | 35.0 | 28.3 | 27.0 | 27.8 |
| All Food | 45.0 | 32.6 | 28.9 | 27.9 |
| Bread and Grains | 36.9 | 32.2 | 30.3 | 30.1 |
| Meats | 63.0 | 54.5 | 45.7 | 32.0 |
| Dairy Products and Eggs | 38.9 | 33.6 | 29.0 | 24.5 |
| Oils | 37.8 | 21.1 | 17.4 | 15.8 |
| Fruits and Nuts | 25.4 | 15.6 | 11.5 | 9.6 |
| Vegetables | 40.0 | 14.2 | 21.1 | 47.6 |
|  | Booshehr (One Intervention District) | | |  |
| All Goods & Services | 38.0 | 28.0 | 26.0 | 27.5 |
| All Food | 51.5 | 35.0 | 31.2 | 29.6 |
| Bread and Grains | 21.8 | 21.0 | 14.6 | 14.7 |
| Meats | 78.3 | 63.2 | 58.8 | 41.6 |
| Dairy Products and Eggs | 41.5 | 32.8 | 27.6 | 25.5 |
| Oils | 71.5 | 39.8 | 23.1 | 19.6 |
| Fruits and Nuts | 22.0 | 15.3 | 9.4 | 9.6 |
| Vegetables | 52.5 | 22.0 | 29.1 | 46.8 |
|  | Tehran (One Intervention District) | | |  |
| All Goods & Services | 34.9 | 30.6 | 29.1 | 29.3 |
| All Food | 46.0 | 37.5 | 32.8 | 30.5 |
| Bread and Grains | 43.1 | 43.2 | 40.9 | 39.9 |
| Meats | 59.7 | 52.5 | 43.7 | 32.3 |
| Dairy Products and Eggs | 48.5 | 42.0 | 35.3 | 29.3 |
| Oils | 29.2 | 19.2 | 16.3 | 15.3 |
| Fruits and Nuts | 21.3 | 16.2 | 12.6 | 7.8 |
| Vegetables | 42.0 | 23.0 | 27.4 | 57.2 |
|  | Semnan (Non-Intervention and One Intervention District) | | | |
| All Goods & Services | 33.8 | 27.7 | 25.2 | 26.2 |
| All Food | 46.3 | 33.2 | 27.0 | 24.1 |
| Bread and Grains | 40.8 | 35.8 | 27.4 | 24.1 |
| Meats | 70.4 | 60.5 | 44.1 | 26.7 |
| Dairy Products and Eggs | 36.6 | 32.5 | 27.8 | 23.3 |
| Oils | 32.1 | 21.6 | 18.8 | 16.5 |
| Fruits and Nuts | 23.6 | 13.3 | 5.5 | 7.5 |
| Vegetables | 41.5 | 6.7 | 21.6 | 44.0 |
