# Supplementary material for: Motivating non-physician health workers to reduce the behavioral risk factors of non-communicable diseases in the community: a field trial study
Source: Arch Public Health. 2023 Mar 10;81:37. doi: 10.1186/s13690-023-01047-w (PMC9998263; doi:10.1186/s13690-023-01047-w)
Supplement: Supplementary file 5 — Additional file 5. IRPONT collaborators. [file 13690_2023_1047_MOESM5_ESM.docx]

**Additional file 5.** IRPONT collaborators:

Ministry of Health and Medical Education of Iran collaborator:

Dr. Mohammad Reza Rahbar

Iran University of Medical Sciences collaborators, Tehran, Iran:

Dr. Jalil Koohpadezadeh, Dr. Babak Eshrati, Dr. Omid Pournik, Dr. Mohammad Hadi Naseh, Dr. Betul Taefi, Dr. Nasir Dehghan, Dr. Neda Soleimanvandi, Ms. Fatemeh Alborzi, Ms. Ruqieh Alizadeh, Mr. Jaafar Alyari, Ms. Leila Bektosan, Ms. Parastoo Bolourian, Ms. Sahar Hassandoost, Ms. Maryam Hassanzadeh, Mr. Mehdi Mansouri, Ms. Rana Mashakhi, Ms. Parvaneh Mirzaei, Ms. Arezo Mohammad-Salahi, Mr. Abolhassan Mohammadian, Mr. Gholamreza Nemati, Mr. Hassan Nicksima, Ms. Zahra Qahri-Mobser, Ms. Raedeh Rezaee, Ms. Susan Salim, Ms. Maryam Shahverdi, Ms. Sara Sufi, Ms. Khadijeh Tandideh

Bushehr University of Medical Sciences collaborators, Bushehr, Iran:

Dr. Abdul Mohammad Khajehean, Dr. Mohammad Mehdi Khajehean, Dr. Razieh Hajioni, Ms. Kobra Aghaee, Ms. Elham Ahmadi-Khorram, Mr. Fatah Allah Hajiani, Ms. Akram Ansari-Far, Ms. Esmat Dehghani, Mr. Mohammad-Sadegh Eskandari, Ms. Zahra fakhar, Mr. Ali Gharibi, Ms. Fatemeh Hayat, Mr. Amin Jahanbakhsh, Mr. Rahim Khademi, Ms. Maria Khishdoost-Borazjani, Ms. Jamileh Moarefi, Ms. Kobra Mohammadi, Mr. Hafiz Omidi, Ms. Azadeh Salemi, Mr. Jahanshir Shahneh, Ms. Homeyra Souleimani, Ms. Mina Tanha, Ms. Marzieh Zaker-Hosseini, Ms. Masoumeh Zamani

Semnan University of Medical Sciences collaborators, Semnan, Iran:

Dr. Jafar Jandaghi, Dr. Mohammad-Naser Rahbar, Ms. Aghababayian Robabeh, Ms. Zahra Aliannejadi, Mr. Mehdi Alikhanian, Mr. Mostafa Amir-Fakhrian, Ms. Atefeh Ayubi-Far, Ms. Zohreh Badami, Ms. Zahra Barati, Ms. Elham Behnam, Ms. Faezeh Binaean, Ms. Parisa Chitsaz, Ms. Sedigheh Darbndi, Ms. Sareh Faraji-Khorshidi, Ms. Neda Ghandali, Ms. Homeyra Ghezlou, Mr. Hassan Ghorbani, Ms. Haniyeh Hassani, Mr. Hassan Jahanshiri, Ms. Zahra kheirkhahan, Mr. Ghorban Mohammad Khojami, Ms. Sara Khorrami, Ms. Shahrbanoo Khorsi-Damghani, Mr. Mohammad-Reza Khoshkam, Mr. Mehdi Majeri, Ms. Arezou Mansourian, Ms. Somayeh Mohammadi, Ms. Razieh Mokhberian, Mr. Ali Niknejad, Ms. Mina Rabiei-Far, Ms. Saeedeh Rezai, Ms. Maryam Roshanaee, Mr. Mahmoud Sabzali, Mr. Ali Sadeghi-Moghaddam, Ms. Nahid Samiie-Rad, Ms. Sabereh Shabiri, Mr. Ahmad Shah-Hosseini, Mr. Mohammad Souri, Mr. Mostafa Talebi, Ms. Masoumeh Touli
